# Usability and feasibility of a tablet-based e-coach for older adults in rehabilitation units to improve nutrition and physical activity: a prospective pilot study

**DOI:** 10.1186/s12877-023-04204-6

**Published:** 2023-09-19

**Authors:** Lisa Happe, Marie Sgraja, Andreas Hein, Vincent Quinten, Rebecca Diekmann

**Affiliations:** 1 Department of Health Services Research, Assistance Systems and Medical Device Technology, Carl von Ossietzky University Oldenburg, Ammerländer Heerstr. 114, Oldenburg, Germany; 2 Department of Health Services Research, Junior research group “nutrition and physical function in older adults”, Carl von Ossietzky University Oldenburg, Ammerländer Heerstr. 114, Oldenburg, Germany

**Keywords:** Older adults, Health behaviour, Nutrition, Physical activity, Rehabilitation, Tablet computers

## Abstract

**Background:**

For older adults (≥ 70 years), it is often challenging to maintain new nutrition and physical activity behaviours learned in rehabilitation. To minimize the risk of negative health consequences when returning home, an e-coach can be helpful. Aligning the program with an established concept such as the Transtheoretical Model of Behaviour Change (TTM) and guidance from healthcare professionals can optimize behaviour change.

**Objective:**

This prospective single-arm pilot study aimed to assess the usability and feasibility of a nutrition and mobility e-coach for older adults during and after rehabilitation for a period of 9 weeks. In addition, we examined the change in the TTM phase as an indicator of the participant’s readiness to change or the changes made.

**Methods:**

Older adults (≥ 70 years) with nutrition deficits and/ or mobility limitations were recruited in a rehabilitation centre. Participants’ phases of behaviour change in the TTM were identified by comparing current nutrition and physical activity habits via self-report with age-specific nutrition and physical activity recommendations. They received a tablet with the e-coach containing educational and interactive elements on the topics of nutrition and physical activity in older age. Participants used the e-coach and received support from healthcare professionals. The TTM phases were assessed at five times; the e-coach content was adjusted accordingly. Usability was assessed using the System Usability Scale (SUS, Score range: 0-100). Timestamps were used to evaluate how frequently participants used the e-coach: high (≥ 67% of the days), medium (66 − 33% of the days), and low (< 33% of the days).

**Results:**

Approximately 140 patients were approached and n = 30 recruited. Complete data sets of n = 21 persons were analysed (38% female, mean age 79.0 ± 6.0 years). The SUS was 78.6 points, 11 participants (42%) were classified as high users, 6 (39%) as medium users and 4 (19%) as low users. After nine weeks, 15 participants (71%) achieved the physical activity recommendations (baseline: 33%, n = 7). Nutrition recommendations were achieved by 14 participants (66%) after nine weeks (baseline: 24%, n = 5).

**Conclusion:**

The e-coach seems to be usable and feasible for older adults. We identified some optimization potentials for our application that can be transferred to the development of comparable e-health interventions for vulnerable older adults.

**Supplementary Information:**

The online version contains supplementary material available at 10.1186/s12877-023-04204-6.

## Background

In rehabilitative care for older patients, malnutrition and limitations in activities of daily living occur more frequently than in the general population of older adults [1]. Malnutrition and functional status (e.g. limitations in activities of daily living) are not only associated with each other but usually occur with other health problems such as sarcopenia or frailty syndrome, which also lead to further comorbidities and severely adverse health outcomes [1–3]. Specific nutritional and physiotherapeutic care often enable an improvement in nutritional status and physical activity and has thus, a positive effect in turn on independence in everyday life [4]. However, health-promoting nutrition and physical activity behaviours need to be maintained after rehabilitation to sustain the improvement in the long term.

To support older people in implementing and maintaining recommendations, strategies, and interventions after discharge, e-health technologies could be useful. The term e-health refers to information and communication technologies that aim to improve the exchange, transmission, networking, and support of healthcare members and healthcare systems [5]. Systematic reviews have already shown positive effects of the use of e-health technologies in the form of apps and fitness trackers on physical activity in community-dwelling older adults [6–8]. Interventions that use behaviour change strategies and programs that are guided by experts seem to be of special benefit [6]. In a recent review by Robert et al. on the effectiveness of nutrition e-health interventions, only two of the 41 included studies involved participants over 70 years of age [7]. One of these studies demonstrated that the use of video consultation significantly reduced the incidence of metabolic complications in community-dwelling older adults with enteral nutrition [9]. In the other study, telemonitoring combined with computer-assisted nutrition education improved nutritional status, adherence to nutrition recommendations, and increased physical activity [10]. A systematic review by Kraaijkamp et al. focused on geriatric rehabilitation and evaluated additionally the feasibility, and usability of e-health interventions. Overall, simple e-health interventions (e.g. health sensors) for which participants received regular support from experts appeared to be feasible for geriatric rehabilitation patients [8].

E-health interventions that targeted the areas of nutrition and physical activity in one program and that were developed for vulnerable patient groups were not identified in these reviews. Accordingly, an e-health intervention in form of an application hereafter referred to as “e-coach”, was developed [11]. The e-coach should provide information on nutrition and exercise in older age in a structured program and support the patient in implementing the recommendations. According to the findings of the reviews mentioned before [6, 8], we used behaviour change techniques when compiling the content of the e-coach. The use of behaviour change techniques was based on the transtheoretical model of behaviour change (TTM) developed by Prochaska and DiClemente. This model assumes that individuals need different stimuli and strategies to change their behaviour, depending on which phase of behaviour they are in. In this context, five phases of behaviour change are distinguished. In the first phase (pre-contemplation), there is no awareness of the problem and a change is not even considered. In the second phase (contemplation), the person is already evaluating whether a behaviour change could be beneficial. In phase three (planning) there is already a concrete plan to change the behaviour. If the person has successfully changed their behaviour, they are in phase four (action) if the behaviour has only been performed for a short time and phase five (maintenance) if the behaviour has been successfully implemented for a longer period. As expert guidance also appears to have positive effects on the applicability and effectiveness of e-health application, applications was designed to be integrated into the care process with nutritional and physical therapy. Accordingly, the modules and its contents were adaptable by the therapists and considered the assessments’ results carried out in patient’s appointments.

The e-coach was developed following the DIN EN ISO 9241 − 210:2019 Ergonomics of human-system interaction—Part 210: Human-centred design of interactive systems and the user-centred design process (German version) [12, 13]. Following these frameworks, the user context was analysed with focus groups and user requirements derived [14]. Screens were designed using Adobe XD and the e-coach was programmed using Java as an Android App optimized for a 10-inch tablet. With three iterative test phases, we enhanced the usability of the e-coach with older adults in an inpatient rehabilitation centre [11]. Detailed information on these steps was published elsewhere [11, 14].

### Objective

This study aimed to evaluate the usability and feasibility of the previously developed nutrition and mobility e-coach application of older adults during and after rehabilitation over a period of 9 weeks.

## Methods

### Study design

This study was a prospective single-arm pilot study with older adults (≥ 70 years) during and after inpatient rehabilitation. Data were collected between August 2021 and June 2022. The participants used a tablet-based e-coach with nutritional and physical activity content. The study was a priori registered in the German Clinical Trials Register (DRKS-ID: DRKS00024481). Ethical approval was obtained from the Ethical Review Board of the Carl von Ossietzky University Oldenburg (registration number: 2021–030).

### Participants and recruitment

Older adults were eligible based on the following inclusion criteria: (1) age ≥ 70 years, (2) able to speak and understand German (3) malnutrition according to the Mini Nutritional Assessment Short Form (MNA-SF) (0–7 points) or at least of one risk factor for malnutrition (weight loss within the last three months, reduced food intake within the last three months, low body mass index (BMI) (< 23 kg/m²)) [15], or (4) presence of at least one sign of reduced mobility (walking speed < 0.8 m/s, Short Physical Performance Battery ≤ 8 points, Timed “Up & Go” Test > 20 s.). Exclusion criteria were (1) severe visual impairment (e.g. inability to read large font on a screen), (2) severe hearing impairment (e.g. deafness), (3) inability to understand study information and provide informed written consent (e.g. aphasia or severe cognitive impairment or dementia), (4) presence of nutrition-associated diseases that require special nutritional recommendations (e.g. short bowel syndrome), (5) participation in other studies aimed at changing nutritional or physical activity behaviour.

Participants were recruited from a rehabilitation centre’s geriatric and cardiology wards. They were identified by an electronic patient database search. These patients were informed about the study in a personal meeting. Additionally, flyers in the patient´s wards informed about the study.

### Assessments and outcome measures

#### Overview

All assessments were conducted by trained study team members (LH and MS) with a professional background in physical therapy or nutritional therapy in face-to-face testing sessions either in the rehabilitation centre or at the participant’s home (t0: baseline, t1: baseline + 1 week, t2: baseline + 2.5 weeks, t3: baseline + 4.5 weeks, t4: baseline + 9 weeks). Depending on the assessment time point (t0-t4) appointment durations were 20 to 50 min. A list of the assessments on the different time points is shown in Table 1.

**Table 1:** Assessments per time point

	t0^a^	t1^b^	t2^c^	t3^d^	t4^e^
Sociodemographic data (age, gender, height, weight)	X		X		X
Mini Nutritional Assessment Short Form	X		X		X
TTM phase nutrition (nutritional history (of breakfast, lunch, dinner in a typical week) + TTM questions)	X	X	X	X	X
TTM phase Physical Activity (Physical Activity Scale for the Elderly (Items 4,5,6) + TTM questions)	X	X	X	X	X
Short Physical Performance Battery	X	X	X	X	X
Timed “Up & Go”	X		X		X
Technology commitment	X				
System Usability Scale					X
Subjective user experience questionnaire (7 Items)					X

^a^t0: Baseline

^b^t1: Baseline + 1 week

^c^t2: Baseline + 2.5 weeks

^d^t3: Baseline + 4.5 weeks

^e^t4: Baseline + 9 weeks

Study data were first documented on paper and then transferred to the REDCap (Research Electronic Data Capture) data management system by two study team members independently from each other [16, 17]. The data were then checked by a third study team member and merged into a final data set.

#### Nutritional data and physical activity

The German version of the MNA-SF (0–14 points) was used to screen for malnutrition and risk factors for malnutrition. Based on six questions, the categories normal nutritional status (12–14 points), risk of malnutrition (8–11 points) and malnutrition (0–7 points) are differentiated [18]. The Short Physical Performance Battery (SPPB) and the Timed “Up & Go” Test (TUG) were conducted to evaluate mobility problems. The SPPB assesses physical functionality with three tasks: static balance, walking speed and time it takes to rise 5 times up from a chair. The rating is divided into four categories: very poor performance (0–3 points), poor performance (4–6 points), moderate performance (7–9 points) and good performance (10–12 points) [19, 20]. The TUG measures the time it takes a person to get up from a chair, walk 3 m, turn around, walk back and sit back down on the chair. If it takes less than 20 s to perform the test, the person is assumed not to be limited in basic transfers [21].

#### The phase of behaviour change

At each time point (t0-t4), we assessed whether the nutrition and physical activity areas could be scored as compliant with the respective minimum requirements.

For the nutrition domain, a total of 6 categories were assessed: (1) cereal products, (2) vegetables and fruits, (3) dairy products, (4) meat, fish, and eggs, (5) fats and oils, (6) drinking amount. For this purpose, a nutritional anamnesis of the last week was conducted with the participants. The reported consumption amounts were compared with the recommendations for people ≥ 65 years of age of the German Nutrition Society. According to the recommendations, the following minimum consumption amounts were matched with the reported consumption amounts: cereal products (at least 4 slices of bread per day and potatoes, rice, or pasta seven days per week), vegetables and fruits (at least 3 servings of vegetables and 2 servings of fruits per day), dairy products (4 servings of milk or dairy products per day), meat, fish, and eggs (at least 3 servings of meat and/or 2 servings of fish, and/or 3 eggs per week (one portion of meat or fish corresponds to about one egg and can be replaced by the same)), fats and oils (at least 1 tablespoon of fat and 1 tablespoon of oil per day), and drinking amount (at least 1500 ml of drinks per day) [22]. If participants were missing only one portion of the respective categories, the recommendations for this category were still considered fulfilled. If at least one of these categories was not met, the nutrition domain was considered not met on the corresponding time point. Participants for whom the nutrition domain was rated as “not fulfilled” were told in which category or categories they did not meet the minimum intake and asked whether they were not thinking about changing their behaviour (pre-contemplation) or thinking about it (contemplation) or already planning the change (planning). Participants who were already achieving the recommendations were asked whether they have been doing this for a short time (action) or for a longer time (maintenance).

For the physical activity domain, the time and frequency of activities were surveyed in three categories: (1) moderate activities, (2) intense activities, (3) strength training. Items 4, 5 and 6 from the Physical Activity Scale for the Elderly questionnaire were used to identify how often and for how long moderate physical activity, vigorous physical activity, and exercises to improve muscle strength were carried out in the past week [23]. This information was compared with the German national physical activity recommendations for people over 65. A minimum of 150 min of moderate physical activity per week or 75 min of vigorous physical activity per week should be performed. In addition, muscle-strengthening exercises should be done at least two days per week [24]. If the recommendations were not achieved, the willingness to change behaviour was asked in the same way as for nutrition (pre-contemplation, contemplation, planning). If the recommendations had already been achieved, the time since this amount of physical activity had been performed was queried again (action or maintenance).

#### Technology commitment

Technology commitment was assessed with a questionnaire with 12 items, which can be answered on a 5-point Likert scale. Thus, a final score between 12 and 60 points can be achieved, and it is possible to calculate a mean value over all 12 items. The items cover the areas of contact, interest, and expectations of the use of technologies [25].

#### Usability

System Usability Scale (SUS) was used to assess the usability of the e-coach. The SUS (0-100 points) is a standardized questionnaire with 10 statements on the usability of a system. To answer the items, a 5-point Likert scale is used. A score above 68 is considered above average and the following score categories were designated by specific adjectives: best imaginable (100 − 84.1 points), excellent (84.0-80.8 points), good (80.7–71.1 points), ok (71.0-51.7 points), poor (51.6–25.1 points), worst imaginable (25 − 0 points) [26, 27].

#### Feasibility

In terms of feasibility, this study focused on practicality (to what extent could the e-coach be used with the intended participants using existing resources and circumstances) and acceptance (to what extent is the e-coach judged suitable and satisfying by the participants) [28].

Timestamps on different screens were evaluated at the end of the study to assess practicability. By evaluating the timestamps, it was possible to track the frequency of use of the e-coach. For this purpose, it was evaluated on how many days during the intervention period activities were recorded on the tablet and the respective percentages of days of use during the intervention period were calculated. No minimum number of days of use per week was imposed on the participants in advance. To evaluate possible different trends in TTMs at different usage frequencies, we divided our study participants into three subgroups based on the percentage of days the e-coach was used over the total study period. For this purpose, e-coach use on ≥ 67% of the days in the intervention period was considered as “high use“, on 66 − 33% of the days were considered as “medium use“, and on < 33% of the days was considered as “low use“.

Acceptance of the e-coach was assessed by a self-developed questionnaire. The questionnaire consisted of seven questions on the relevance of the content, use of the e-coach in everyday life, ease of use of the e-coach, and functionality and was conducted at t4. Each statement was rated on a scale with two antonyms between 1 and 5, where 1 expressed the highest agreement and 5 the highest disagreement (e.g. the information from the app is useful ① ② ③ ④ ⑤ useless for my everyday life).

#### Intervention

The participants received a tablet (Lenovo YT-X705L, Lenovo Group Limited, Quarry Bay, Hongkong, China) with the preinstalled e-coach. The e-coach was individually adjusted by a study member according to the results of the participant’s TTM phase (for nutrition and physical activity respectively). Different elements such as videos, texts, or quizzes, were used to provide the information. In addition, the e-coach included active input options, such as a nutrition diary with feedback on nutrient and drinking quantity and an exercise diary for the documentation of performed physical exercises with an overview of the achievement of goals. The content of the modules and the interactive elements in the area of nutrition, were based on the recommendations of the European Society for Clinical Nutrition and Metabolism guidelines on clinical nutrition and hydration in geriatrics [29] and the recommendations of the German Nutrition Society (DGE) on eating and drinking in old age [30]. Content and recommendations on physical activity were based on the national recommendations on exercise and physical activity promotion [24] and the information from the initiative “Älter werden in Balance” (“Getting Older in Balance”) program of the Federal Center for Health Education and the recommendations from the IN FORM initiative run by Bundesarbeitsgemeinschaft der Seniorenorganisationen (German National Association of Senior Citizens’ Organizations) [31]. The content of the modules and the target values of the interactive elements considered the requirements of the respective nutrition-associated diseases and the level of exercise was also adapted to the physical status of the person. The basic functions of the tablet and the e-coach were explained by the study team members and a user manual with photos of possible interactions was handed out. Together with the participants, the study team member selected which content might be particularly relevant or interesting and practised how to access this content. If participants wanted to use the exercise diary (possible in the planning, action and maintenance phases in TTM physical activity), a target was set for the minimum number of days the exercises should be performed.

At each assessment time point, the TTM phase has been evaluated and the e-coach was adapted to the new TTM phase according to the results. These visits also included a consultation with a study team member with a professional background in physical therapy or nutritional therapy. In these consultations, questions and experiences about handling the e-coach, nutrition and physical activity topics and evaluations of the nutrition and exercise diary were discussed and together identified which contents and actions would also be beneficial for the participant. The time points were arranged in a way that personal meetings took place more frequently at the beginning and decreased in frequency in the course of the intervention. In case of difficulties in dealing with the technology or increased need for advice, it was possible to arrange further consultation appointments. In addition, the participants could contact the study team at any time in case of difficulties.

### Data analysis

Statistical analyses were conducted in R version 4.2.1 (The R Project for Statistical Computing, Vienna, Austria) and SPSS version 27.0 (SPSS Inc., Chicago, IL, USA). Only data sets with data for all five time points were analysed.

Descriptive statistics (absolute and relative frequencies, median, and standard deviation) were used to analyse the assessment data. In addition, using the three user groups (high, medium, low), the trajectories of the TTM nutrition and physical activity phases at the five time points and the SUS score were analysed descriptively and compared.

The variables BMI, MNA-SF, SPPB and TUG were exploratively tested for significance between t0 and t4. In case of normal distribution, a t-test for paired samples was conducted. A sign test was calculated for ordinally scaled data and if there was no normal distribution. If interval-scaled, non-normally distributed data were given, a Wilcoxon signed-rank test was calculated.

## Results

The study involved a total of 30 participants. In the course of the nine weeks, 8 participants dropped out of the study due to the following reasons: medical problems (n = 2), feeling stressed (n = 2), private issues (n = 2), problems with handling the e-coach (n = 1), experienced no benefit of the use (n = 1). Due to a technical malfunction of one tablet, access to the data was not possible and could therefore not be included in the analysis. Accordingly, 21 complete data sets were included. The mean age of the participants was 79.0 (± 6.0) years, 38% (n = 8) participants were female and the mean technology commitment was 40.3 (± 7.8) with a mean score across the 12 items of 3.0 ± 0.7 points. A comparison of the nutrition and physical activity characteristics collected at baseline (t0) and after nine weeks (t4) is shown in Table 2.

**Table 2:** Comparison of nutritional and physical activity data at baseline and after 9 weeks, n = 21

Characteristic	t0^a^	t4^b^	P-value
BMI kg/m^2^, mean (SD)	26.3 ± 3.8	27.3 ± 3.4	0.000
female, mean (SD)	25.3 (3.1)	26.4 (3.6)	
male, mean (SD)	26.9 (4.1)	27.9 (3.2)	
MNA-SF, mean (SD)	9.1 ± 2.1	11.1 ± 2.4	0.019
malnutrition, n (%)	5 (24)	2 (10)	
risk for malnutrition, n (%)	13 (62)	8 (38)	
normal, n (%)	3 (14)	11 (52)	
SPPB, mean (SD)	9.1 ± 2.1	11.1 ± 2.4	0.019
0–3 points (very poor performance), n (%)	1 (5)	1 (5)	
4–6 points (poor performance), n (%)	7 (33)	4 (19)	
7–9 points (moderate performance), n (%)	10 (48)	9 (43)	
10–12 points (good performance), n (%)	3 (14)	7 (33)	
TUG Test in seconds, mean (SD)	16.0 (7.5)	15.5 (9.9)	0.013
TUG Test > 20 s	8 (38)	6 (29)	

^a^t0: baseline

^b^t4: baseline + 9 week

Abbreviations: BMI kg/m^2^: Body Mass Index kg/m^2^, MNA-SF: Mini Nutritional Assessment Short Form, SPPB: Short Physical Performance Battery, TUG: Timed “Up & Go”

### Feasibility (user groups and subjective e-coach rating)

**Fig. 1:**
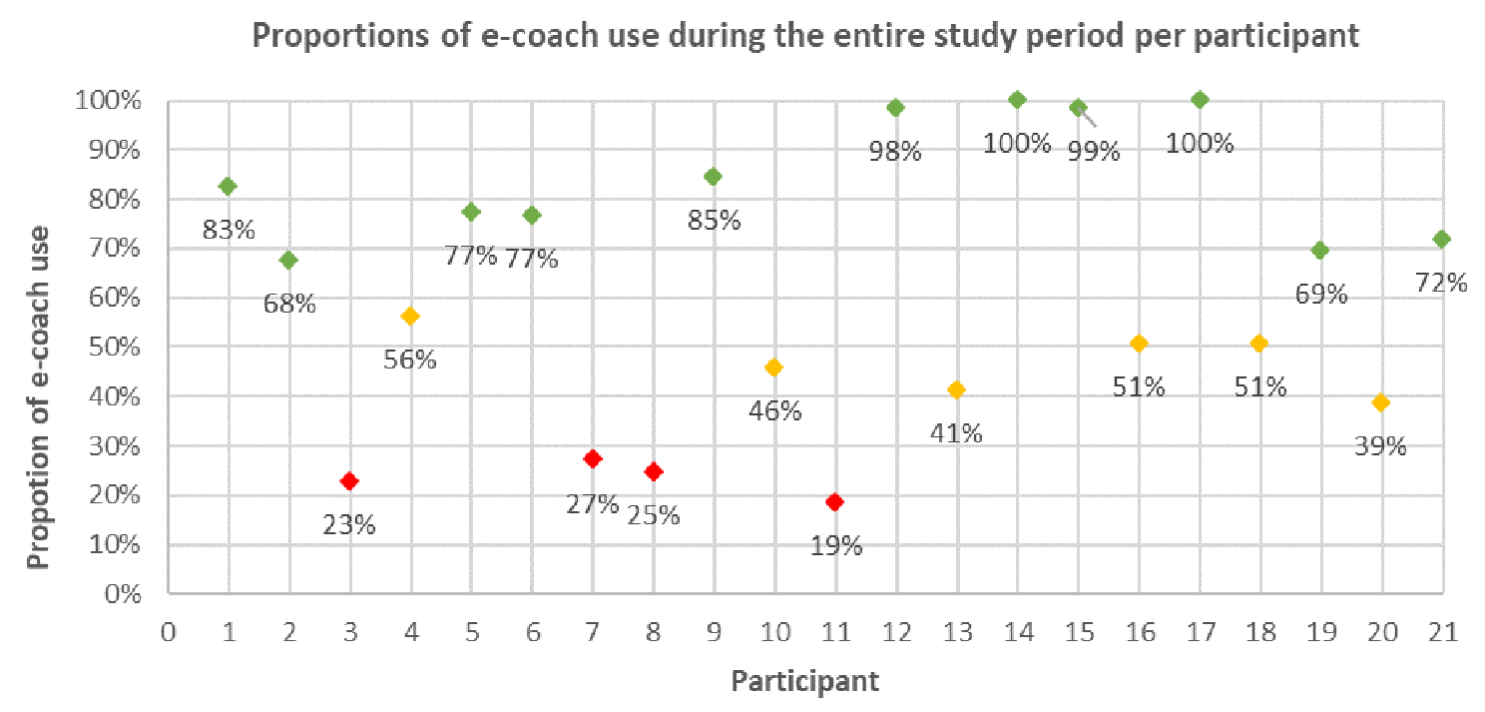
Percentage of days of use of the e-coach during the intervention period per participant. The use categories are marked in different colours. Green = percentage of use ≥ 67% (high), yellow = percentage of use 66 − 33% (medium) and red = percentage of use < 33% (low)

The average intervention period was 65 (± 5.8) days. The participants used the e-coach at an average of 40.1 (± 17.7) days in this timespan. Classified into the three user groups, 52% (n = 11) showed high use, 29% (n = 6) medium use and 19% (n = 4) low use. A diagram of the individual amount of use of the e-coach per participant is shown in Fig. 1.

**Fig. 2:**
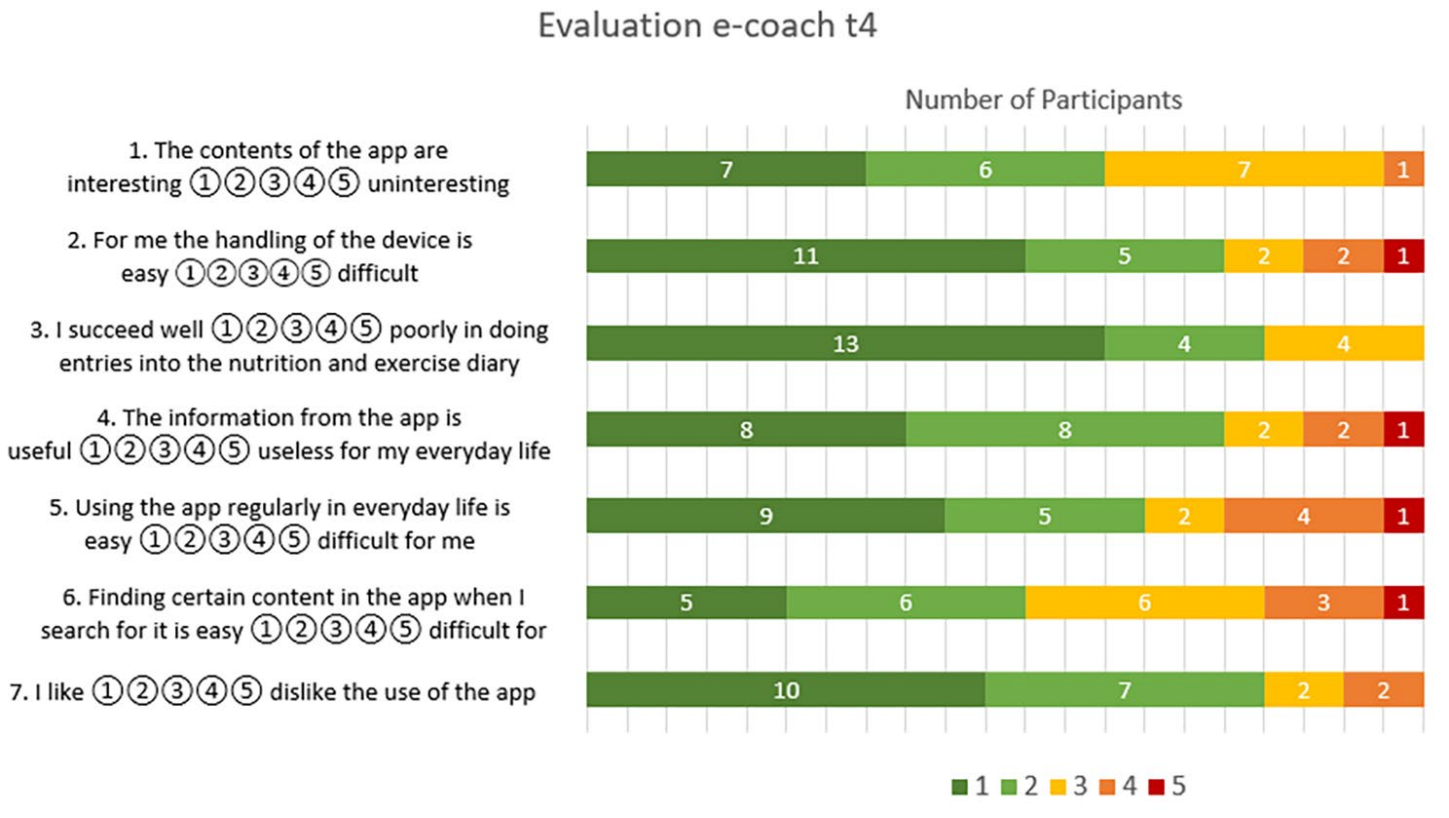
Subjective assessment regarding different aspects of the use of the e-coach

The evaluation of the e-coach content, interaction with the e-coach and integrability in the everyday life of the participants was predominantly positive. A detailed presentation of the responses to the questions is shown in Fig. 2.

### Usability

**Fig. 3:**
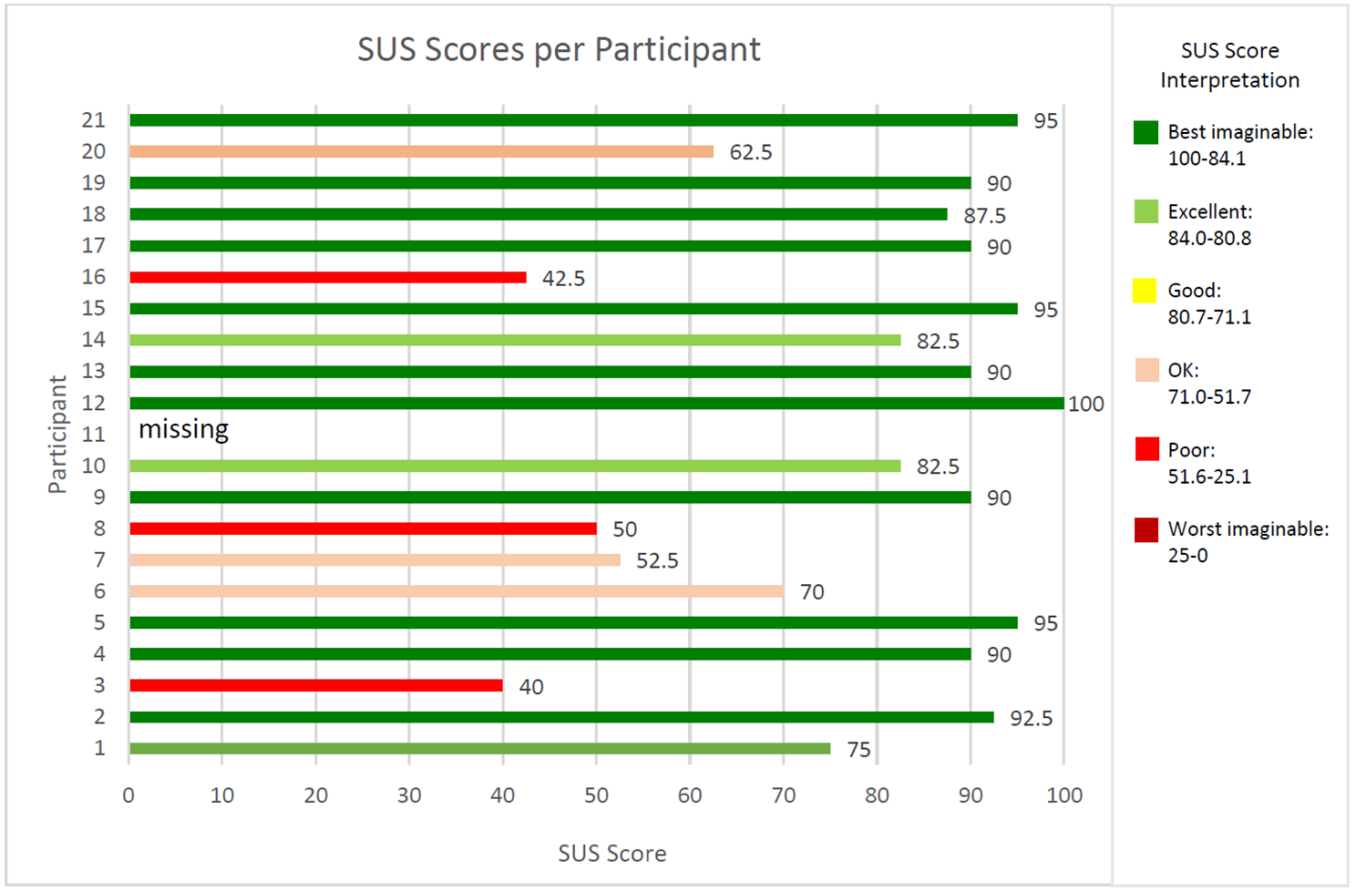
System Usability Scale (SUS) score per participant (n = 21). Interpretation of the scores with descriptions by adjectives is shown on the right. The SUS score is missing for participant 11

The SUS of the e-coach was rated by the participants with an average of 78.6 (± 18.1). Participants with a high frequency of use rated the SUS with an average of 88.1 (± 8.2), participants with a medium frequency of use with 75.8 (± 17.7) and participants with a low frequency of use with 47.5 (± 5.4). A diagram of the SUS score per participant is shown in Fig. 3.

### TTM phase

At baseline, 7 participants (33%) achieved the physical activity recommendations (action phase 24%, n = 5; maintenance phase 10%, n = 2). Most participants did not achieve the recommended amount of strengthening exercise per week at t0 (48%, n = 10). Nutrition recommendations were achieved by 5 participants (24%) at t0 (action phase 14%, n = 3; maintenance phase 10%, n = 2). The categories of vegetables and fruits, dairy products, and drinking amount were most frequently not achieved (38%, n = 8 each).

After 9 weeks (t4), 15 participants (71%) achieved the physical activity recommendations (action phase 14%, n = 3; maintenance phase 57%, n = 12). The physical activity and muscle strengthening exercise categories were not achieved by 3 participants (14%). Nutrition recommendations were achieved by 14 participants (66%) at t4 (action phase 14%, n = 3; maintenance phase 52%, n = 11). The vegetable and fruit (n = 4, 19%) and drinking amount (n = 3, 14%) categories were most frequently not fulfilled. Changes in the TTM phase at t0-t4 are shown in Table 3. A table with the number of individuals not achieving the different categories at t0-t4 is provided in the Appendix (Appendix 1).

**Table 3:** Change in distribution of the individual TTM phases at the five time points (t0-t4)

TTM phase^a^	t0^b^ n (%)	t1^c^ n (%)	t2^d^ n (%)	t3^e^ n (%)	t4^f^ n (%)
**TTM phase physical activity**					
pre-contemplation	1 (5)	0 (0)	2 (10)	0 (0)	0 (0)
contemplation	1 (5)	0 (0)	1 (5)	1 (5)	1 (5)
planning	12 (57)	6 (29)	10 (48)	8 (38)	5 (24)
action	5 (24)	12 (57)	5 (24)	5 (24)	3 (14)
maintenance	2 (10)	2 (10)	3 (14)	6 (29)	12 (57)
missing	0 (0)	1 (5)	0 (0)	1 (5)	0 (0)
**TTM phase nutrition**					
pre-contemplation	2 (10)	1 (5)	1 (5)	1 (5)	1 (5)
contemplation	1 (5)	1 (5)	2 (10)	0 (0)	2 (10)
planning	13 (62)	12 (57)	8 (38)	7 (33)	4 (19)
action	3 (14)	6 (29)	5 (24)	6 (29)	3 (14)
maintenance	2 (10)	0 (0)	5 (24)	7 (33)	11 (52)
missing	0 (0)	1 (5)	0 (0)	0 (0)	0 (0)

^a^TTM phase: phase of the transtheoretical model of behaviour change

^b^t0: Baseline

^c^T1: Baseline + 1 week

^d^T2: Baseline + 2.5 weeks

^e^T3: Baseline + 4.5 weeks

^f^T4: Baseline + 9 weeks

**Fig. 4:**
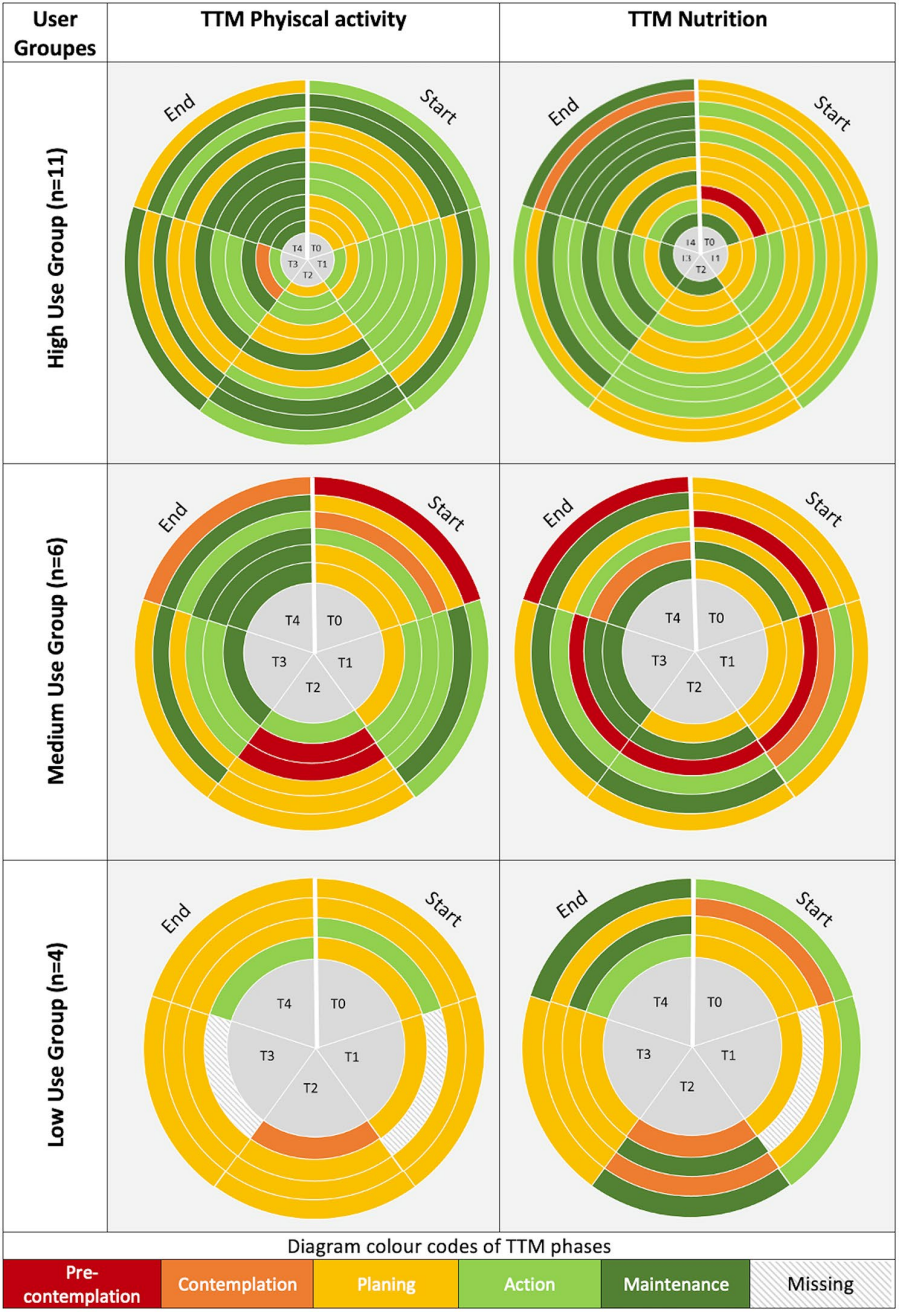
Colour-coded illustration of the different TTM phases per participant and per time point (t0-t4). Participants are subdivided according to the intensity of use of the e-coach into the high, medium or low use group. Each ring represents a participant and each section of the ring represents the TTM phase on the respective time point

Variation in TTM phases regarding the three user types (high, medium, low) is shown by graphs with different colours per TTM phase in Fig. 4.

At t4, a higher proportion of high users (82%, n = 9; vs. t0 46%, n = 5) and medium users (83%, n = 5; vs. t0 17%, n = 1) achieved the physical activity recommendations than low users (25%, n = 1; vs. t0 25%, n = 1). Nutritional recommendations were reached by 8 high users (73%) (vs. t0: 27%, n = 3), 3 medium users (50%) (vs. t0: 17%, n = 1), and 3 low users (75%) (vs. t0: 25%, n = 1) at t4.

## Discussion

Good usability and feasibility of the e-coach was shown among older adults during and after rehabilitation. With a mean SUS score of 78.6, the e-coach achieved a similar score as after the previous iterative optimizations [11]. Other e-health applications used with older adults showed comparable SUS scores [32–34]. The good SUS results are also reflected in the subjective evaluation of the e-coach. Of the 21 participants, 17 (81%) stated that they liked the e-coach. The evaluation of practicability based on the amount of use of the e-coach also shows indications of good feasibility with 11 participants (52%) in the high use group and 6 participants (29%) in the medium use group.

However, the wide range of the SUS score (100 − 40 points) indicates that there is still potential for improvement. Overall, 5 participants (23%) rated the e-coach with a score below the average SUS score of 67 established by Bangor et al. [27]. The participants who rated the e-coach below 67 points were all in the low or medium user group, which may indicate that individuals who were less proficient with the e-coach also used it less. Further, these individuals had a lower technology commitment (31.8) than the overall study population (40.3). Most participants (81%, n = 17) rated their ability to use the nutrition and exercise diary with 1 (62%, n = 13) or 2 (19%, n = 4), which corresponded to the highest two agreement ratings. The general handling of the device was also rated as easy by most participants (76%, n = 16) with 1 (52%, n = 11) or 2 (24%, n = 5). However, the evaluation of the item “Finding certain content in the app when I search for it is easy ①②③④⑤ difficult for me” shows that almost half of the participants (48%) still see a need for improvement here. A potential beneficial feature could be the integration of a search function. The ability to integrate the e-coach into the participants’ everyday lives is also an important aspect of acceptance. Regular use of the e-coach in everyday life was rated as easy by two-thirds of participants (67%, n = 14), with 9 participants (43%) selecting the highest level of agreement. However, 5 participants (24%) also indicated that it was rather difficult. This corresponds roughly to the proportion of participants who were in the low use category. Additionally, it must be considered that 2 participants who dropped out reported stress as a reason. An often-mentioned factor in the integration of technologies into everyday life for older people is often that the use should be associated with low time requirement [14, 35]. Therefore, features such as automatic tracking of exercise via sensors or faster documentation of meals in the nutrition diary (e.g. with image recognition or a “standard meal” if the same foods are always eaten for breakfast) could be useful optimizations.

The changes in physical activity behaviour in the TTM demonstrate positive developments in most participants in comparison between t0 and t4. A study by Silveira et al. also used a tablet-based app with older adults and evaluated behaviour change with the TTM phases. The study demonstrated that after 12 weeks, 21 participants (64%) reached the action or maintenance phase [36]. Some differences between our study and the study by Silveira et al. can be observed. While in our study more participants (57%, n = 12) were in the planning phase at t0 compared to 4 participants (n = 12) in Silveira et al., there were already 15 participants (46%) in the maintenance phase at t0 while in our study only 2 participants (10%) had this phase at t0. These differences could be because different study populations were included. In the study by Silveira et al., persons from institutions for older adults (e.g. a day-care centre) were cantered while we recruited inpatient rehabilitation patients. Individuals from rehabilitation have mostly previously experienced an adverse health event that affected their physical activity level and often results in inability to an maintain their previous physical activity routine. By participating in a rehabilitation program, it is also expected that the person plans to increase their performance. We noticed in our study that at t1, 14 participants (67%) achieved the physical activity recommendations, but at t2, only 8 participants (38%) still did. One possible interpretation is the method used to assess physical activity. At t1, participants had been in rehabilitation for more than a week and were receiving a structured exercise program that they did not have to schedule themselves. At t2, however, many participants had already been discharged and were back at home. It is possible, therefore, that the loss of the exercise routine from rehabilitation and other daily duties meant that many participants no longer achieved the exercise recommendations at t2. At t3, 11 (52%) and at t4 15 participants (71%) managed to achieve the physical activity recommendations.

The classification into TTM phases was based on the achievement or non-achievement of the dietary and physical activity recommendations. Accordingly, how this was assessed should also be discussed. We compared the age-specific nutrition and physical activity recommendations based on self-report with the food consumed in the last week and the type and amount of physical activity. Self-reports always have the disadvantage of being affected by bias [37, 38]. Other methods, such as the use of activity sensors to collect physical activity or the use of a weighing protocol or photographs of the plate before and after eating, would enable a more objective recording [37–39]. Since the participants were in their own homes for most of the study period and monitoring their diet would have meant a major intrusion into their privacy and into the independence that the e-coach was supposed to promote, a nutritional history of the last week was taken. In the context of physical activity behaviour change using the TTM, there are several studies which also use self-reports to evaluate physical activity [36, 40, 41].

If the change in the TTM phases is evaluated under consideration of the three user categories, it can be observed that more high (82%, n = 9) and medium (83%, n = 5) than low users (25%, n = 1) achieved the physical activity recommendations at t4. This could be related to the fact that the use of the exercise diary already implies a certain frequency of use days to document the exercises performed. In addition, the educational content also provided participants with information e.g. on the effects of exercise in older age or recommendations for setting physical activity goals. Participants who used the e-coach more frequently may have gained correspondingly more information about the benefits and opportunities of physical activity. Interestingly, both high and medium users showed a positive development of their TTM phases. This could indicate that increased use does not lead to greater change, but also that other factors related to the intervention, such as support from the study team, probably contribute in part to the changes. The aim of the e-coach should be to support sustainable behaviour change. The exercises and educational content were designed to educate the participants and support them in developing routines. Our study indicates that this was also possible for participants from the medium user group. These findings are comparable to a study by Bickmore et al. where participants used an embodied conversational agent (ECA) for two months, which was designed to increase the daily step count. Participants in the intervention group used the ECA on 60% of the days during the study period and significantly increased their number of steps compared with the control group [42].

The number of participants who achieved the nutritional recommendations increased from five participants (24%) at t0 to 14 (66%) at t4. A change in the proportion of individuals who achieved the nutritional recommendations at t1 as compared to t2 could not be determined here. At t1, six participants (29%) achieved the recommendations and at t2, there were 10 participants (48%). This may be related to the fact that participants were again more independent to choose what they wanted to eat after discharge and they were back in their usual environment. A closer look at the food categories that participants did not adequately consume at t4 indicates potentials for improvement in the content of the e-coach. Of the seven participants (33%) who did not reach nutritional recommendations at the end of the study, four (57%) did not reach the “vegetables and fruits” category. To date, the e-coach content has focused more on protein intake and other macronutrients. These nutrients are considered to be of particular importance in the treatment and prevention of sarcopenia, and it is well-known inadequate protein intake is prevalent in many older people [29, 43]. However, vegetables and fruits are important components of a balanced diet in older age, as they provide fibre and micronutrients such as vitamins. For example, a deficiency in micronutrients can also lead to an increased risk of developing frailty [43]. A study by van Doorn-van Atten et al. shows that an increase in vegetable and fruit consumption is possible through a technical support system. In the study, computer-tailored nutrition education for older people in combination with visits by a trained healthcare professional was compared with a control group [44]. The addition of further educational elements on vegetable and fruit consumption could therefore be a useful extension of the e-coach. Furthermore, 3 of the 7 participants who did not reach the nutritional recommendations at t4 were found to drink insufficient amounts of fluids. If fluid intake is inadequate, this can have many negative consequences for older individuals e.g. increased risk of delirium and mortality [45]. Content on the relevance of sufficient fluid intake was addressed in the e-coach through more educational elements than vegetable and fruit consumption. In addition, participants who used the food diary could also see the previous amount of drinks entered in the drinking protocol. Since our study participants were free to choose what content they viewed, it might be helpful if certain content, such as the fluid intake strategies were suggested again by the program to those who have a hydration deficit. In a study with patients with coronary heart disease, dietary recommendations were sent as text messages at certain intervals. Using this specific reminder of relevant recommendations increased adherence to the given nutritional guidelines in the study compared with a control group [46]. In our study, we also referred to relevant content individually during the appointments with participants. However, since “Finding certain content in the app when I search for it is easy ①②③④⑤ difficult for me” was the lowest rated item in the e-coach’s subjective evaluation, a push notification that leads directly to the relevant content could be useful. Another possibility would be for participants who use the food diary to receive a reminder in the e-coach when no drinks have been documented for a longer period was already stated as useful by the experts in the focus groups for identifying relevant content for the e-coach, but was not implemented due to a lack of personnel and time resources [14].

### Limitations

The major limitation of our study is the limited ability to interpret the results due to the small sample size and the lack of a control group. Since all participants received nutritional and physical therapy interventions through rehabilitation in addition to using the e-coach, we are unable to differentiate whether the behaviour change occurred through our e-coach intervention, the support by the study team members or the rehabilitation interventions. The calculated significance values must also be considered under these conditions and do not allow conclusions to be made about effects of our intervention. When collecting physical activity and food consumption data to compare with age-specific recommendations, a response bias is possible due to the use of self-reports. In future studies, it would be useful to measure these parameters with more objective methods, such as activity sensors, in order to improve the robustness of the results. Because of limited staff and technical resources on the part of the study team and also limited time availability of potential participants due to rehabilitation activities, study participants were included at different points in their rehabilitation process. When we compare the change in high and medium users to low users, we can observe some indications of improvements in TTM physical activity through greater use of the e-coach, but because the samples are so small, these trends must be considered very cautiously. Furthermore, the gender distribution does not correspond to that of the population over 65 years of age in Germany. While in the general population about 56% are female in this age group, in our study it is only 38% [47]. Possible reasons could be a higher interest or experience with technology among older men compared to older women [48]. However, older men are often underrepresented in studies on improving health-promoting behaviours and are generally less likely to take advantage of health behaviour improvement offers [49]. Accordingly, the e-coach could also provide an opportunity for older men to feel more motivated to accept support for longer-term changes in nutrition and physical activity behaviour. In general, we must assume a selection bias, because more individuals with a greater interest in nutrition, physical activity, and technology participated in our study. This is also confirmed by the fact that only a few participants were in the precontemplation or contemplation phase at baseline. The educational background of the participants was not surveyed as part of this study. It is not possible to assess the influence of this parameter, either in terms of a selection bias of the study population or a possible association between educational background and skills in using the e-coach. In general, the evidence on the influence of educational background on the use of health information technology is currently inconclusive [50]. Since we are addressing the feasibility of the e-coach, the high drop-out rate in our study of 27% (n = 8) is also an important limitation. At least 4 of the 8 individuals who dropped out of the study stated that they terminated participation due to stress (n = 2), problems in dealing with the technology (n = 1), and because no benefits were perceived from its use (n = 1). This suggests that the e-coach should be further optimized so that its use is even easier and can be better integrated into the everyday lives of older people.

## Conclusion

The study showed indications for good usability and feasibility of the e-coach for older adults during and after a rehabilitation stay under the supervision of experts. The participants’ usage behaviour indicates that the older adults were able to use the tablet and the e-coach well, and the subjective feedback also shows positive evaluations of the e-coach. It was also possible to find optimization potentials for the e-coach, which could improve the usability and also feasibility for even more participants. To our knowledge, it is the first e-coach that was developed specifically with and for older inpatient rehabilitation patients to further support nutrition and physical activity behaviour also after rehabilitation. On this basis, a larger study with more participants and a control group can be planned to assess the impact of the use of the e-coach on nutrition and physical activity behaviour change.

### Electronic supplementary material

Below is the link to the electronic supplementary material.

Supplementary Material 1

## Data Availability

The datasets used and/or analysed is this study are available from the corresponding author upon reasonable request.

## Abbreviations

BMI: Body Mass Index
ECA: Embodied conversational agent
MNA-SF: Mini Nutritional Assessment Short Form
REDCap: Research Electronic Data Capture
SPPB: Short Physical Performance Battery
SUS: System Usability Scale
TTM: Transtheoretical Model of Behaviour Change
TUG: Timed “Up & Go” Test
