# Supplementary material for: Usability and feasibility of a tablet-based e-coach for older adults in rehabilitation units to improve nutrition and physical activity: a prospective pilot study
Source: BMC Geriatr. 2023 Sep 19;23:578. doi: 10.1186/s12877-023-04204-6 (PMC10507984; doi:10.1186/s12877-023-04204-6)
Supplement: Supplementary file 1 — Supplementary Material 1 [file 12877_2023_4204_MOESM1_ESM.docx]

| **Recommendations** | | **T0** | | **T1** | | **T2** | | **T3** | | **T4** | |
| --- | --- | --- | --- | --- | --- | --- | --- | --- | --- | --- | --- |
|  | | n  (%) | %  total ^h^ | n  (%) | %  total ^h^ | n  (%) | %  total ^h^ | n  (%) | %  total ^h^ | n  (%) | %  total ^h^ |
| **Not achieving physical activity (PA) recommendations^a^** | | 14 (67) | 71 | 7 (33) | 33 | 13 (62) | 62 | 10 (48) | 48 | 6 (29) | 29 |
|  | <150 min moderate PA per week or <75 min vigorous PA per week | 6 (40) | 29 | 1 (14) | 5 | 5 (39) | 24 | 3 (30) | 14 | 3 (50) | 14 |
|  | Strength training at <2 days per week | 10 (71) | 48 | 6 (86) | 29 | 8 (62) | 38 | 9 (90) | 43 | 3 (50) | 14 |
| **Not achieving nutritional recommendations^a,b^** | | 16 (76) | 76 | 14 (67) | 67 | 11 (52) | 52 | 8 (38) | 38 | 7 (33) | 33 |
|  | <4 slices of bread per day^c^ and/or <7 days per week potatoes, rice or pasta | 0 (0) | 0 | 2 (14) | 10 | 5 (46) | 24 | 2 (25) | 10 | 1 (14) | 5 |
|  | <3 servings of vegetables and/or <2 servings of fruit per day^d^ | 8 (50) | 38 | 7 (50) | 33 | 3 (27) | 14 | 5 (63) | 24 | 4 (57) | 19 |
|  | <4 portions of milk and dairy products per day^e^ | 8 (50) | 38 | 5 (36) | 24 | 6 (55) | 29 | 3 (38) | 14 | 2 (29) | 10 |
|  | <3 servings of meat and/or < 2 servings of fish and/or <3 eggs per week^f^ | 5 (31) | 24 | 2 (14) | 10 | 2 (18) | 10 | 2 (25) | 10 | 0 (0) | 0 |
|  | <1 tablespoon of fat and/or <1 tablespoon of oil per day | 1 (6) | 5 | 2 (14) | 10 | 1 (9) | 5 | 0 (0) | 0 | 0 (0) | 0 |
|  | Drinking amount <1500 ml^g^ | 8 (50) | 38 | 3 (21) | 14 | 2 (18) | 10 | 2 (25) | 10 | 3 (43) | 14 |
| ^a^ Difference from the number of participants who did not reach the recommendations possible, because some participants did not reach more than one category.  ^b^ In the different categories, the recommendations were still marked as achieved even if 1 portion was missing.  ^c^ 1 slice of bread can also be replaced by 2 tablespoons of cereals.  ^d^ A glass of fruit or vegetable juice can replace one serving of fruit or vegetables.  ^e^ One serving is equivalent to 100 ml of yogurt or milk, 50 g of cream cheese or a slice of cheese.  ^f^ One portion of meat or fish corresponds to about one egg and can be replaced by the same.  ^g^ For persons with drinking volume restrictions, the drinking volume prescribed by the physician is considered the drinking amount target.  ^h^% of total study sample (n=21). | | | | | | | | | | | |

Appendix 1. Nutritional and physical activity recommendation categories not achieved by participants at the measurement time points (t0-t4).
